# Expectations of non-COVID-19 deaths during the pre-vaccine pandemic: a process-control approach

**DOI:** 10.1186/s12889-022-14829-8

**Published:** 2023-01-23

**Authors:** Ralph Catalano, Joan A. Casey, Alison Gemmill, Tim Bruckner

**Affiliations:** 1 School of Public Health, University of California, Berkeley, CA 94720 USA; 2 Department of Environmental Health Sciences, Columbia University Mailman School of Public Health, New York, USA; 3 Department of Population, Family and Reproductive Health, Johns Hopkins Bloomberg School of Public Health, Baltimore, USA; 4 Program in Public Health and Center for Population, Inequality and Policy, University of California, Irvine, USA

**Keywords:** COVID-19, All-cause mortality, Germany

## Abstract

**Background:**

Debate over “social distancing” as a response to the pandemic includes the claim that disrupting clinical and public health programming dependent on human-to-human contact increased non-COVID-19 deaths. This claim warrants testing because novel pathogens will continue to emerge. Tests, however, appear frustrated by lack of a convention for estimating non-COVID-19 deaths that would have occurred had clinical and public health programming during the pre-vaccine pandemic remained as efficacious as in the pre-pandemic era. Intending to hasten the emergence of such a convention, we describe and demonstrate “new-signal, prior-response expectations” suggested by research and methods at the intersection of epidemiology and process control engineering.

**Methods:**

Using German data, we estimate pre-pandemic public health efficacy by applying Box-Jenkins methods to 271 weekly counts of all-cause deaths from December 29 2014 through March 8 2020. We devise new-signal, prior-response expectations by applying the model to weekly non-COVID-19 deaths from March 9 2020 through December 26 2020.

**Results:**

The COVID-19 pandemic did not coincide with more non-COVID-19 deaths than expected from the efficacy of responses to pre-pandemic all-cause deaths.

**Conclusions:**

New-signal, prior-response estimates can contribute to evaluating the efficacy of public health programming in reducing non-COVID-19 deaths during the pre-vaccine pandemic.

**Supplementary Information:**

The online version contains supplementary material available at 10.1186/s12889-022-14829-8.

## Background

Impeding human-to-human contact as a public health response to pre-vaccine COVID-19 deaths has proved controversial for several reasons. These include the claim that “social distancing” significantly reduced the efficacy of life-saving clinical and public health programs that require human-to-human contact [1]. Debate over this claim will likely persist because novel infectious pathogens will continue to emerge [2]. Agreeing a convention for estimating the association between social distancing and non-COVID 19 deaths would seem, therefore, an important task for epidemiologists. We suggest and demonstrate an essential component of such a convention – the estimation of non-COVID-19 deaths that would have occurred had clinical and public health programming during the pre-vaccine pandemic remained as efficacious as in the pre-pandemic era. We refer to these counterfactual deaths as “expectations.” Detectable differences between these expectations and intra-pandemic, pre-vaccine non-COVID-19 deaths would gauge shifts, if any, in the efficacy of clinical and public health programming [3].

We make several assumptions when deriving our expectations of death. First, populations suffer mortality proportional to, among other phenomena, how quickly public health agencies and clinicians learn from their attempts to minimize deaths. Second, public health agencies monitor the incidence of death and develop expectations of future incidence [3]. Third, deaths greater than expected trigger a public health and clinical response based on prevailing explanations of the processes that affect population health. And last, these explanations, and the interventions they shape, presumably change based on the inferred efficacy of prior interventions.

Because the above assumptions describe behavior studied under the rubric of “process control engineering,” we borrow terms and arguments from that field to describe and demonstrate our approach to estimating expected deaths [4, 5]. We, for example, use “signal” to refer to the incidence of all-cause death before, and non-COVID-19 deaths after, the onset of the pandemic. We use “response” to refer to clinical and public health interventions begun when that signal exceeded expectations. And, as described below, we use process-control modeling to derive expectations of death.

Process control assumes that “autocorrelation” in a signal, or how quickly unexpected values return to expected levels, gauges the efficacy of response [5]. The argument against social distancing as a public health strategy presumes that autocorrelation in non-COVID-19 deaths during the pre-vaccine pandemic appeared longer than that among pre-pandemic all-cause deaths (i.e., non-COVID-19 deaths in the pre-pandemic period). In other words, an unexpectedly high count of weekly non-COVID-19 deaths during the pre-vaccine pandemic would presumably persist into more subsequent weeks than would a similarly unexpected and high count of all-cause deaths in weeks before the pandemic. This presumption implies that applying the best-fitting model of autocorrelation in pre-pandemic all-cause deaths to non-COVID-19 deaths during the pre-vaccine pandemic will estimate fewer deaths during the pre-vaccine era than observed. We refer to these estimated deaths as “new-signal, prior-response expectations” because they result from applying autocorrelation in pre-pandemic all-cause deaths (i.e., prior response) to observed intra-pandemic, pre-vaccine non-COVID-19 deaths (i.e., new signal).

## Methods

### Data

We derive new-signal, prior-response expectations with data from Germany during the 42 intra-pandemic, pre-vaccine weeks defined by the first COVID-19 death (i.e., March 9, 2020) and the first vaccinations (i.e., December 26, 2020). We chose Germany for our example because its pandemic policies affected a relatively large population and because its income and age distributions appear close to those of Europe as a whole [6]. Scholarly controversy, moreover, remains in Germany over how much the pre-vaccine intervention of impeding human-to-human contact affected non-COVID-19 deaths [7, 8]. We do not, however, claim that the results of our demonstration will generalize elsewhere.

All-cause death data described below came from the Human Mortality Database, a publicly and freely available source of life table data that meet standards of completeness and accuracy set by demographers not involved in this paper [9]. COVID-19 deaths came from the Our World in Data publicly available website [10]. Attributing death to SARS-CoV-2 infection can involve judgement subject to error [11]. Data from Germany, however, appear relatively accurate [11].

### Analyses

We derived new-signal, prior-response expectations in 4 steps described below.

1. We created a time-series of non-COVID-19 deaths in Germany for 313 Monday-through-Sunday weeks starting December 29, 2014 and ending December 26, 2020. We used all-cause deaths for pre-pandemic values, and COVID-19 deaths subtracted from all-cause deaths for the 42 intra-pandemic, pre-vaccine weeks. Five years of pre-pandemic weekly counts provide sufficient information to identify potential seasonality.
2. We used well-developed and widely applied process control methods, pioneered by Box and Jenkins [5], to identify autocorrelation in 271 weekly counts of all-cause deaths in the pre-pandemic period defined as December 29, 2014 through March 8, 2020. Box and Jenkins offered a general theory of autocorrelation, a common notation for models describing patterns in time-series data, and, most important, rules for determining which models best describe autocorrelation in an observed set of serial measurements. Models identified and subsumed by Box-Jenkins methods include, for example, the “reproduction number” estimates epidemiologists commonly use to describe outbreaks of communicable disease [12]. Box and Jenkins models have, moreover, successfully fit and predicted intra-pandemic morbidity [13].
  The general form of a Box-Jenkins model, estimated with maximum likelihood methods, applied to weekly non-COVID-19 deaths in the 271 pre-pandemic weeks, is as follows:
  $$\left(1-\upphi \textrm{B}\right)\left(1-\Phi {\textrm{B}}^{\textrm{p}}\right){\Delta}^{\textrm{d}}\left({\textrm{Z}}_{\textrm{t}}\right)=\textrm{C}+\left(1-\uptheta \textrm{B}\right)\left(1-\Theta {\textrm{B}}^{\textrm{q}}\right){\textrm{a}}_{\textrm{t}}$$
  where Z_t_ is the count of non-COVID-19 deaths in week t. C is a constant. a_t_ is the value, at week t, of independently and normally distributed counts of weekly deaths unexpected from autocorrelation in the observed series (i.e., Z). B is the “backshift operator” that equal the values of Z and a at weeks t-1, t-p, and t-q. Δ^d^ is a “differencing operator” used to remove a secular trend (or sine wave) by taking differences at week t-d. φ and θ are, respectively, autoregressive and moving average parameters that estimate the fraction of Z_t_ added to, or subtracted from, Z at t + 1. Φ and Θ are, respectively, higher order (e.g., seasonal) autoregressive and moving average parameters that estimate the fraction of Z_t_ added to, or subtracted from, Z at t + p or t + q in which p and q are greater than 1.
  Not all series will exhibit autocorrelation “fit” with a constant as well as autoregressive, moving average, and differencing terms. Indeed, the contributions of the Box-Jenkins approach include rules for identifying needed terms and how far into the future they project proportions of Z [5]).
3. We applied the model and coefficients estimated in step 2 to the entire 313 weeks of observed data. The fitted values for the 42 intra-pandemic, pre-vaccine weeks serve as our new-signal, prior-response expectations. These estimate weekly non-COVID-19 deaths in a hypothetical Germany practicing social distancing, but in which the efficacy of the response of clinicians and public health agencies equals the efficacy of their response to all-cause deaths before the pandemic. The residuals of this model fitting (i.e., observed deaths less expectations for the entire series) gauge the degree to which deaths differed from those expected under the assumption of equivalent efficacy.
4. In our last step we answered the question: did the pre-vaccine response of German public health agencies to the COVID-19 pandemic coincide with detectably more non-COVID-19 deaths than expected from the efficacy of their response to pre-pandemic all-cause deaths? We did so, first, by determining whether the mean of the 42 intra-pandemic, pre-vaccine residuals from the model fitted in step 3 fell outside the 95% confidence interval of the mean (i.e., 0) of the 271 pre-pandemic residuals. If social distancing decreased the efficacy of policies that reduced non-COVID-19 deaths, the mean of these 42 residuals would fall above the interval. Second, we determined whether autocorrelation in the 42 intra-pandemic, pre-vaccine weeks differed from that in the 271 pre-pandemic weeks. We tested for such a difference by computing the autocorrelation function of the 42 residuals through 36 lags and applying the Ljung-Box [13] test of autocorrelation. If social distancing increased autocorrelation in non-COVID-19 deaths, this test would detect autocorrelation.

## Results

**Fig. 1:**
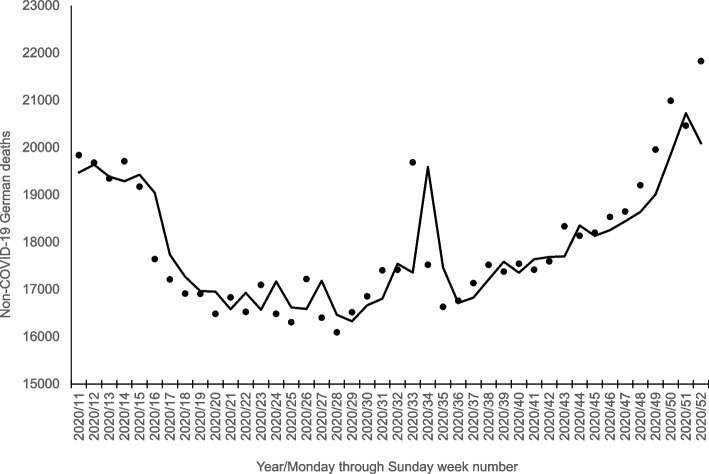
Non-COVID-19 German deaths (points) and new-signal, prior-response expectations (line) for 42 intra-pandemic, pre-vaccine weeks (i.e., 3/9/2020 through 12/26/2020)

Non-COVID-19 deaths in Germany over the 313-week test period (i.e., December 29, 2014 through December 26, 2020) ranged from 15,233 to 26,777 with a mean of 17,928. Non-COVID-19 deaths in the 42 pre-vaccine pandemic weeks, shown as points in Fig. 1, ranged from 16,091 to 21,825 with a mean of 17,940.

Step 2 of our demonstration yielded the following Box-Jenkins model of the 271-week pre-pandemic series.

$$\left(1-0.897\textrm{B}\right)\left(1+0.155{\textrm{B}}^{26}\right)\left({\textrm{Z}}_{\textrm{t}}-17926\right)={\textrm{a}}_{\textrm{t}}$$

in which Z_t_ is weekly all-cause deaths in week t and 17,926 is the mean of Z. The 0.897 (standard error = 0.028) autoregressive parameter indicates that high or low values in week t persisted into week t + 1. The 0.155 (standard error = 0.059) autoregressive parameter indicates that high or low values at week t “echoed” at week t + 26. The residuals of the model (i.e., a_t_) exhibited no autocorrelation and a mean of 0. No differencing (i.e., Δ^d^ in the general model) or moving average (i.e., θB^q^ in the general model) parameters were needed to model autocorrelation in the series.

Step 3, applying the model shown above to all 313 weeks of data, yielded expected (i.e., fitted) values of which the last 42 serve as our new-signal, prior-response expectations for the pre-vaccine pandemic weeks. These values appear as the line in Fig. 1.

The last step in our analyses yielded no support for the claim that social distancing in Germany increased non-COVID-19 deaths above those expected from the efficacy of pre-pandemic clinical and public health policies. The mean of the 42 intra-pandemic, pre-vaccine residuals was 54.747 with a standard error of 114 implying no detectable difference from 0 and, therefore, no shift in the level of these deaths. The Ljung-Box test [14], moreover, detected no autocorrelation in the residuals (i.e., Q = 32.6 with 36 degrees of freedom). Indeed, 36 lags of the autocorrelation function included no coefficient twice its standard error.

Based on the logic described above, we conclude that the pre-vaccine response of German clinicians and public health agencies to the COVID-19 pandemic did not coincide with detectably more non-COVID-19 deaths than expected from the efficacy of their response to pre-pandemic deaths.

## Discussion

We offer a rationale and method for devising expectations needed to answer the question: did a population’s response to intra-pandemic deaths coincide with more non-COVID-19 deaths than expected from the efficacy of its response to all-cause deaths *before* the pandemic? These new-signal, prior-response expectations led us to answer that, for example, the response by German public health agencies and clinicians, which included impeded human-to-human contact, did *not* coincide with greater non-COVID-19 deaths than expected from the efficacy of their response to pre-pandemic all-cause deaths.

We acknowledge that deaths shown in Fig. 1 appear to drift *below* expected during 5 weeks of “shutdown” (i.e., weeks 16 through 20) and above expected later in the year when public resistance to social-distancing policies increased. We note, however, that social-distancing policies, albeit of varying stringency, applied throughout the 42 weeks and that, as our test results imply, the sum of differences between expected and observed non-COVID-19 deaths over the 42 weeks did not differ from expected. We, therefore, would resist the *post-hoc* suspicion that impeding human-to-human contact reduced non-COVID-19 deaths.

Artifacts of the data we used may have affected our results. Defining COVID-19 deaths remains, as noted above, subject to human judgement and error. We note, however, that accounting policies did not change during our test period implying that systematic errors unlikely affected temporal variation in our series. Our data will also fail to capture non-COVID-19 deaths that occurred after the test period but may have been averted had human to human contact been greater during the pre-vaccine pandemic.

We could repeat our analyses for sub-categories of mortality and would likely find some for which the new-signal, same-response expectations appeared less than the observed values implying that social distancing may have reduced the efficacy of pre-pandemic interventions. We note, however, that reconciling that result with our main finding would logically require discovering causes of death for which the expectation appeared greater than the observed value. Social distancing, in other words, would have decreased the likelihood of some other cause or causes of death.

We used process-control logic and methods to answer the question: did Germany’s social distancing policies coincide with more non-COVID-19 deaths than expected from the efficacy of its response to all-cause deaths *before* the pandemic? The “pandemic studies” literature includes many attempts to answer a related but fundamentally different question: did the pandemic coincide with more deaths than expected from history [15]? Answering this question requires estimating deaths that process-control logic would characterize as “same-signal, same-response” expectations because neither deaths nor policies during the pandemic era affect their derivation. Researchers have derived “same-signal, same-response” expectations using several methods, including “stacked calendars” [16, 17] and exponential smoothing [18], drawn from the forecasting literature. These approaches would not serve our purposes because answering our question requires expectations affected by deaths observed during the pandemic (i.e., that react to a new signal). Unlike Box-Jenkins methods, moreover, neither “stacked calendars” nor exponential smoothing was intended to identify and exploit all forms of autocorrelation. Expectations based on them may not, therefore, reflect the efficacy of pre-pandemic clinical and public health policies in reducing autocorrelation in deaths (i.e., not fully capture the pre-pandemic response).

## Conclusions

We borrowed constructs and methods from the process control literature because they help connect our arguments to the debate surrounding the use of public health regulation to manage epidemics. Process control assumes a change in response when sensors detect signals above a “set point” or largest acceptable value. In our example, all cause deaths in the early weeks of the pandemic exceeded Germany’s set point and triggered a response, like those elsewhere, intended to impede human-to-human contact. That intervention proved controversial in part due to the claim that impeding social interaction increased non-COVID-19 deaths. Using new-signal, prior-response estimates, we find no support for that claim.

We intended our analyses of the German experience to demonstrate the new-signal, prior-response method. Our findings may not, therefore, generalize to other populations. Indeed, we offer the new-signal, prior-response approach so that public health authorities can assess the efficacy of programming that likely varied in effect given differences in vulnerabilities, values, and resources among the populations served.

## Supplementary Information

**Additional file 1.**

## Data Availability

All data used in this study are publicly and freely available at Human Mortality Database. Short-term Mortality Fluctuations (STMF) data series. https://www.mortality.org/ or Ritchie H, Mathieu E, Rodés-Guirao L, Appel C, Giattino C, et al. Coronavirus Pandemic (COVID-19). 2020. https://ourworldindata.org/coronavirus
