# Supplementary material for: Expectations of non-COVID-19 deaths during the pre-vaccine pandemic: a process-control approach
Source: BMC Public Health. 2023 Jan 23;23:155. doi: 10.1186/s12889-022-14829-8 (PMC9870657; doi:10.1186/s12889-022-14829-8)
Supplement: Supplementary file 1 — Additional file 1. [file 12889_2022_14829_MOESM1_ESM.docx]

***SUPPLEMENTARY INFORMATION***

**THE PC SCA STATISTICAL SYSTEM ( Release 5.2-Enterprise )**

**COPYRIGHT 1985-1998, SCIENTIFIC COMPUTING ASSOCIATES. ALL RIGHTS RESERVED**

**SCA PRODUCT IDENTIFICATION: UTS & EXPERT (GSA OPTIONAL)**

**SCA SOFTWARE IDENTIFICATION: UC-BERKELEY.NETW ( 98110618 )**

**RELEASE DATE: 9/ 1/97**

**SIZE OF WORKSPACE IS 999000 SINGLE PRECISION WORDS**

**DATE -- 9/17/** TIME -- 10:17:40**

**--**

***Nc19d is the weekly counts of Non-COVID19 deaths in Germany for 313 Monday-through-Sunday weeks starting December 29, 2014 and ending December 26, 2020.***

**--**

**Des nc19d.**

**VARIABLE NAME IS NC19D**

**NUMBER OF OBSERVATIONS 313**

**NUMBER OF MISSING VALUES 0**

**STATISTIC STD. ERROR STATISTIC/S.E.**

**MEAN 17928.2939 103.4930 173.2320**

**VARIANCE 3352478.4069**

**STD DEVIATION 1830.9774**

**C. V. .1021**

**SKEWNESS 1.4522 .1378**

**KURTOSIS 2.8417 .2747**

**QUARTILE**

**MINIMUM 15233.0000**

**1ST QUARTILE 16613.5000**

**MEDIAN 17427.0000**

**3RD QUARTILE 18884.5000**

**MAXIMUM 26777.0000**

**RANGE**

**MAX - MIN 11544.0000**

**Q3 - Q1 2271.0000**

**Sel old nc19d. New nc19ds. span (1,271)**

**VARIABLE NC19D IS EDITED, THE RESULT IS STORED IN VARIABLE NC19DS**

**VARIABLE NC19DS IS A 271 BY 1 MATRIX**

**--**

**New nc19ds**

***nc19ds is the weekly counts of Non-COVID19 deaths in Germany for 271 weeks from December 29, 2014 through March 8, 2020***

**VARIABLE NAME IS NC19DS**

**NUMBER OF OBSERVATIONS 271**

**NUMBER OF MISSING VALUES 0**

**STATISTIC STD. ERROR STATISTIC/S.E.**

**MEAN 17926.5351 114.7502 156.2223**

**VARIANCE 3568421.7830**

**STD DEVIATION 1889.0267**

**C. V. .1054**

**SKEWNESS 1.4718 .1480**

**KURTOSIS 2.8216 .2949**

**QUARTILE**

**MINIMUM 15233.0000**

**1ST QUARTILE 16584.0000**

**MEDIAN 17427.0000**

**3RD QUARTILE 18883.5000**

**MAXIMUM 26777.0000**

**RANGE**

**MAX - MIN 11544.0000**

**Q3 - Q1 2299.5000**

**Uid nc19ds. Max 60**

**NAME OF THE SERIES . . . . . . . . . . NC19DS**

**TIME PERIOD ANALYZED . . . . . . . . . 1 TO 271**

**MEAN OF THE (DIFFERENCED) SERIES . . . 17926.5400**

**STANDARD DEVIATION OF THE SERIES . . . 1885.5380**

**T-VALUE OF MEAN (AGAINST ZERO) . . . . 156.5113**

**AUTOCORRELATIONS**

**1- 12 .91 .82 .73 .63 .53 .42 .33 .25 .18 .10 .04 -.02**

**ST.E. .06 .10 .12 .14 .15 .15 .16 .16 .16 .16 .16 .16**

**13- 24 -.08 -.13 -.18 -.21 -.24 -.27 -.28 -.30 -.32 -.35 -.39 -.42**

**ST.E. .16 .16 .16 .16 .17 .17 .17 .17 .17 .17 .18 .18**

**25- 36 -.45 -.47 -.47 -.46 -.45 -.43 -.40 -.37 -.35 -.32 -.28 -.24**

**ST.E. .18 .19 .19 .20 .20 .20 .21 .21 .21 .21 .22 .22**

**37- 48 -.19 -.15 -.10 -.04 .01 .07 .12 .19 .24 .28 .33 .37**

**ST.E. .22 .22 .22 .22 .22 .22 .22 .22 .22 .22 .22 .22**

**49- 60 .41 .44 .48 .50 .50 .50 .49 .47 .42 .37 .32 .25**

**ST.E. .23 .23 .23 .24 .24 .24 .25 .25 .25 .26 .26 .26**

**PARTIAL AUTOCORRELATIONS**

**1- 12 .91 -.07 -.07 -.06 -.12 -.09 .02 .01 -.04 -.06 -.02 -.03**

**ST.E. .06 .06 .06 .06 .06 .06 .06 .06 .06 .06 .06 .06**

**13- 24 -.11 .00 -.02 .00 -.08 .03 -.06 -.02 -.11 -.13 -.11 .00**

**ST.E. .06 .06 .06 .06 .06 .06 .06 .06 .06 .06 .06 .06**

**25- 36 -.12 .03 .04 -.07 -.05 .01 -.02 -.12 -.02 -.01 .05 -.09**

**ST.E. .06 .06 .06 .06 .06 .06 .06 .06 .06 .06 .06 .06**

**37- 48 .06 -.05 -.03 .07 -.03 .06 -.00 .13 -.02 -.00 .03 .03**

**ST.E. .06 .06 .06 .06 .06 .06 .06 .06 .06 .06 .06 .06**

**49- 60 .00 .12 .05 -.01 .00 .04 .00 -.03 -.08 -.03 .01 -.04**

**ST.E. .06 .06 .06 .06 .06 .06 .06 .06 .06 .06 .06 .06**

**--**

***The following 2 model fittings are those used to arrive at the final model (i.e., model1) for the pre-pandemic series (i.e., steps 1 through 3 described in the manuscript Methods sections).***

**Uts nam mod1a. Mod (1)nc19d(cen)=noise**

**Ues mod mod1a. Hol resi(mod1ar) fit (mod1af). span 1,271**

**THE FOLLOWING ANALYSIS IS BASED ON TIME SPAN 1 THRU 271**

**NONLINEAR ESTIMATION TERMINATED DUE TO:**

**RELATIVE CHANGE IN (OBJECTIVE FUNCTION)**0.5 LESS THAN .1000D-02**

**SUMMARY FOR UNIVARIATE TIME SERIES MODEL -- MOD1A**

**-----------------------------------------------------------------------**

**VARIABLE TYPE OF ORIGINAL DIFFERENCING**

**VARIABLE OR CENTERED**

**NC19D RANDOM CENTERED NONE**

**-----------------------------------------------------------------------**

**PARAMETER VARIABLE NUM./ FACTOR ORDER CONS- VALUE STD T**

**LABEL NAME DENOM. TRAINT ERROR VALUE**

**1 NC19D AR 1 1 NONE .9152 .0247 37.03**

**EFFECTIVE NUMBER OF OBSERVATIONS . . 270**

**R-SQUARE . . . . . . . . . . . . . . .835**

**RESIDUAL STANDARD ERROR. . . . . . . .765977E+03**

**--**

**Uid mod1ar. max 60**

**NAME OF THE SERIES . . . . . . . . . . MOD1AR**

**TIME PERIOD ANALYZED . . . . . . . . . 2 TO 271**

**MEAN OF THE (DIFFERENCED) SERIES . . . 2.9866**

**STANDARD DEVIATION OF THE SERIES . . . 765.9709**

**T-VALUE OF MEAN (AGAINST ZERO) . . . . .0641**

**AUTOCORRELATIONS**

**1- 12 .06 .06 .03 .08 .03 -.07 -.07 -.02 .02 -.04 -.05 .04**

**ST.E. .06 .06 .06 .06 .06 .06 .06 .06 .06 .06 .06 .06**

**13- 24 -.06 -.04 -.07 .01 -.08 -.02 -.05 .04 .05 .01 -.09 .04**

**ST.E. .06 .06 .06 .06 .06 .06 .06 .06 .06 .06 .06 .06**

**25- 36 -.11 -.14 -.05 -.03 -.09 -.07 .02 -.04 -.01 -.10 .03 -.07**

**ST.E. .06 .07 .07 .07 .07 .07 .07 .07 .07 .07 .07 .07**

**37- 48 .00 -.02 -.09 .07 -.05 .04 -.06 .06 .06 .01 .03 .04**

**ST.E. .07 .07 .07 .07 .07 .07 .07 .07 .07 .07 .07 .07**

**49- 60 .01 .02 .11 .10 .05 .07 .10 .17 .05 .02 .06 .09**

**ST.E. .07 .07 .07 .07 .07 .07 .07 .07 .07 .07 .07 .07**

**PARTIAL AUTOCORRELATIONS**

**1- 12 .06 .05 .03 .08 .02 -.08 -.07 -.01 .03 -.02 -.03 .05**

**ST.E. .06 .06 .06 .06 .06 .06 .06 .06 .06 .06 .06 .06**

**13- 24 -.07 -.04 -.06 .02 -.08 .00 -.03 .04 .04 .01 -.11 .03**

**ST.E. .06 .06 .06 .06 .06 .06 .06 .06 .06 .06 .06 .06**

**25- 36 -.14 -.14 -.00 -.01 -.08 -.05 .03 -.09 -.03 -.12 .04 -.14**

**ST.E. .06 .06 .06 .06 .06 .06 .06 .06 .06 .06 .06 .06**

**37- 48 .01 -.03 -.13 .00 -.09 -.02 -.13 .03 -.01 -.02 -.01 .01**

**ST.E. .06 .06 .06 .06 .06 .06 .06 .06 .06 .06 .06 .06**

**49- 60 -.11 .00 .04 .03 -.01 .05 .06 .11 .04 .01 .05 .08**

**ST.E. .06 .06 .06 .06 .06 .06 .06 .06 .06 .06 .06 .06**

**--**

**Uts nam mod1. Mod (1)(26)nc19d(cen)=noise**

**Ues mod mod1. Hol resi(mod1r) fit (mod1f). span 1,271**

**THE FOLLOWING ANALYSIS IS BASED ON TIME SPAN 1 THRU 271**

**NONLINEAR ESTIMATION TERMINATED DUE TO:**

**RELATIVE CHANGE IN (OBJECTIVE FUNCTION)**0.5 LESS THAN .1000D-02**

**SUMMARY FOR UNIVARIATE TIME SERIES MODEL -- MOD1**

**-----------------------------------------------------------------------**

**VARIABLE TYPE OF ORIGINAL DIFFERENCING**

**VARIABLE OR CENTERED**

**NC19D RANDOM CENTERED NONE**

**-----------------------------------------------------------------------**

**PARAMETER VARIABLE NUM./ FACTOR ORDER CONS- VALUE STD T**

**LABEL NAME DENOM. TRAINT ERROR VALUE**

**1 NC19D AR 1 1 NONE .8977 .0283 31.69**

**2 NC19D AR 2 26 NONE -.1545 .0599 -2.58**

**EFFECTIVE NUMBER OF OBSERVATIONS . . 244**

**R-SQUARE . . . . . . . . . . . . . . .847**

**RESIDUAL STANDARD ERROR. . . . . . . .737029E+03**

**--**

**Uid mod1r. out prin (lbq). max 60**

**NAME OF THE SERIES . . . . . . . . . . MOD1R**

**TIME PERIOD ANALYZED . . . . . . . . . 28 TO 271**

**MEAN OF THE (DIFFERENCED) SERIES . . . -11.6432**

**STANDARD DEVIATION OF THE SERIES . . . 736.9370**

**T-VALUE OF MEAN (AGAINST ZERO) . . . . -.2468**

**AUTOCORRELATIONS**

**1- 12 .04 .09 .01 .08 .05 -.07 -.05 -.04 .03 -.04 -.06 .07**

**ST.E. .06 .06 .06 .06 .07 .07 .07 .07 .07 .07 .07 .07**

**Q .4 2.4 2.5 4.3 5.0 6.4 7.1 7.5 7.8 8.2 9.3 10.4**

**13- 24 -.10 -.03 -.10 .02 -.08 -.05 -.08 .02 .08 .03 -.08 .04**

**ST.E. .07 .07 .07 .07 .07 .07 .07 .07 .07 .07 .07 .07**

**Q 12.9 13.1 15.7 15.8 17.4 18.1 20.0 20.1 22.0 22.3 24.1 24.5**

**25- 36 -.11 .01 -.06 -.02 -.06 -.04 .04 -.06 -.02 -.12 .00 -.09**

**ST.E. .07 .07 .07 .07 .07 .07 .07 .07 .07 .07 .07 .07**

**Q 28.1 28.2 29.0 29.1 30.3 30.8 31.3 32.2 32.3 36.1 36.1 38.5**

**37- 48 .00 -.02 -.11 .08 -.07 .08 -.08 .08 .03 .04 .05 .03**

**ST.E. .07 .07 .07 .07 .07 .07 .07 .07 .08 .08 .08 .08**

**Q 38.5 38.6 42.2 44.2 45.5 47.7 49.5 51.5 51.7 52.3 53.0 53.4**

**49- 60 -.01 .02 .09 .12 .04 .08 .07 .19 .07 .02 .04 .08**

**ST.E. .08 .08 .08 .08 .08 .08 .08 .08 .08 .08 .08 .08**

**Q 53.4 53.5 55.8 60.5 61.0 63.0 64.6 76.2 77.8 77.9 78.3 80.6**

**PARTIAL AUTOCORRELATIONS**

**1- 12 .04 .09 .00 .08 .05 -.09 -.06 -.03 .04 -.03 -.05 .08**

**ST.E. .06 .06 .06 .06 .06 .06 .06 .06 .06 .06 .06 .06**

**13- 24 -.11 -.05 -.07 .03 -.07 -.04 -.06 .03 .07 .04 -.10 .02**

**ST.E. .06 .06 .06 .06 .06 .06 .06 .06 .06 .06 .06 .06**

**25- 36 -.15 -.00 -.01 -.02 -.05 -.05 .05 -.08 -.05 -.11 .02 -.14**

**ST.E. .06 .06 .06 .06 .06 .06 .06 .06 .06 .06 .06 .06**

**37- 48 .04 -.04 -.10 .04 -.07 .01 -.13 .06 -.01 .03 -.02 .03**

**ST.E. .06 .06 .06 .06 .06 .06 .06 .06 .06 .06 .06 .06**

**49- 60 -.11 .00 .06 .08 -.00 .06 .07 .15 .06 -.01 .04 .10**

**ST.E. .06 .06 .06 .06 .06 .06 .06 .06 .06 .06 .06 .06**

**--**

***The following step applies Model1 to the entire 313 weeks of data as described in step 4 in the Methods section of the manuscript.***

**Fil mod mod1. Old nc19d. New nc19df.**

**THE FOLLOWING ANALYSIS IS BASED ON TIME SPAN 1 THRU 313**

**SERIES NC19D IS FILTERED USING MODEL MOD1 , THE RESULT IS IN NC19DF**

**--**

**Nc19dx=nc19d-nc19df**

***Nc19dx are the expected values resulting from applying Model1 to the entire 313 weeks of data. The last 42 values of NC19dx (listed at the end of this supplement) are our new-signal, same-response expectations for the intra-pandemic, pre-vaccine weeks.***

**Sel old nc19df. New testset. Span (272,313)**

**VARIABLE NC19DF IS EDITED, THE RESULT IS STORED IN VARIABLE TESTSET**

**VARIABLE TESTSET IS A 42 BY 1 MATRIX**

**--**

***TESTSET is the last 42 values of NC19df or residuals need for the last step of our test.***

**Des testset.**

**VARIABLE NAME IS TESTSET**

**NUMBER OF OBSERVATIONS 42**

**NUMBER OF MISSING VALUES 0**

**STATISTIC STD. ERROR STATISTIC/S.E.**

**MEAN 54.7476 113.9918 .4803 [Not different from 0]**

**VARIANCE 545753.2901**

**STD DEVIATION 738.7512**

**C. V. 13.4938**

**SKEWNESS .2592 .3654**

**KURTOSIS 2.0881 .7166**

**QUARTILE**

**MINIMUM -2064.4980**

**1ST QUARTILE -335.2233**

**MEDIAN 44.0725**

**3RD QUARTILE 338.5314**

**MAXIMUM 2329.2830**

**RANGE**

**MAX - MIN 4393.7810**

**Q3 - Q1 673.7546**

***As seen above the mean of testset is not different from 0 implying no difference between the pre- and intra-pandemic values.***

**Uid testset. Out prin (lbq). Max 36.**

**NAME OF THE SERIES . . . . . . . . . . TESTSET**

**TIME PERIOD ANALYZED . . . . . . . . . 1 TO 42**

**MEAN OF THE (DIFFERENCED) SERIES . . . 54.7476**

**STANDARD DEVIATION OF THE SERIES . . . 729.9035**

**T-VALUE OF MEAN (AGAINST ZERO) . . . . .4861**

**AUTOCORRELATIONS**

**1- 12 -.11 .09 .07 .03 .02 -.06 .23 -.08 -.06 .18 -.10 .13**

**ST.E. .15 .16 .16 .16 .16 .16 .16 .17 .17 .17 .17 .17**

**Q .5 .9 1.2 1.2 1.2 1.4 4.1 4.5 4.7 6.6 7.1 8.2**

**13- 24 -.14 -.03 -.09 -.05 .05 -.08 .31 -.03 -.03 -.01 -.03 -.03**

**ST.E. .18 .18 .18 .18 .18 .18 .18 .19 .19 .19 .19 .19**

**Q 9.5 9.6 10.1 10.3 10.5 11.0 18.9 19.0 19.1 19.1 19.2 19.3**

**25- 36 -.10 .03 -.04 -.09 .03 -.11 -.03 -.10 -.10 -.10 -.02 -.09**

**ST.E. .19 .20 .20 .20 .20 .20 .20 .20 .20 .20 .20 .20**

**Q 20.5 20.6 20.8 21.8 22.0 23.7 23.8 25.9 27.8 30.0 30.1 32.6**

**PARTIAL AUTOCORRELATIONS**

**1- 12 -.11 .08 .09 .04 .01 -.07 .21 -.03 -.11 .16 -.06 .11**

**ST.E. .15 .15 .15 .15 .15 .15 .15 .15 .15 .15 .15 .15**

**13- 24 -.12 -.15 -.07 .01 .00 -.01 .32 .05 -.04 -.12 -.05 -.04**

**ST.E. .15 .15 .15 .15 .15 .15 .15 .15 .15 .15 .15 .15**

**25- 36 -.04 -.11 -.01 .00 -.09 -.10 -.11 .02 -.02 -.04 .05 -.08**

**ST.E. .15 .15 .15 .15 .15 .15 .15 .15 .15 .15 .15 .15**

**--**

***As seen above, Testset exhibits no autocorrelation implying that observed intra-pandemic Non-COVID19 deaths exhibited the same autocorrelation as pre-pandemic all cause deaths.***

**Prin years,weeks,nc19d,nc19dx.**

**YEARS IS A 313 BY 1 VARIABLE**

**WEEKS IS A 313 BY 1 VARIABLE**

**NC19D IS A 313 BY 1 VARIABLE**

**NC19DX IS A 313 BY 1 VARIABLE**

***The following lists shows observed Non-COVID19 deaths and our new-signal, same-response expectations for the 42 intra-pandemic, pre-vaccine weeks shown in Figure 1 of the manuscript.***

**YEAR WEEK OBSERVED EXPECTATIONS**

**2020 11 19837 19471.133**

**2020 12 19679 19636.996**

**2020 13 19344 19383.115**

**2020 14 19709 19285.215**

**2020 15 19171 19424.598**

**2020 16 17640 19040.758**

**2020 17 17211 17732.535**

**2020 18 16911 17269.004**

**2020 19 16905 16964.701**

**2020 20 16482 16951.598**

**2020 21 16830 16579.721**

**2020 22 16523 16925.654**

**2020 23 17094 16569.605**

**2020 24 16482 17166.635**

**2020 25 16305 16617.441**

**2020 26 17218 16585.709**

**2020 27 16402 17181.391**

**2020 28 16091 16461.928**

**2020 29 16516 16325.864**

**2020 30 16853 16663.746**

**2020 31 17403 16802.473**

**2020 32 17415 17541.283**

**2020 33 19685 17355.717**

**2020 34 17521 19585.498**

**2020 35 16630 17461.178**

**2020 36 16759 16712.859**

**2020 37 17131 16824.867**

**2020 38 17518 17206.805**

**2020 39 17375 17584.061**

**2020 40 17542 17352.826**

**2020 41 17415 17636.498**

**2020 42 17592 17684.424**

**2020 43 18333 17697.244**

**2020 44 18135 18349.291**

**2020 45 18199 18130.861**

**2020 46 18531 18252.840**

**2020 47 18645 18438.436**

**2020 48 19203 18636.479**

**2020 49 19955 19006.588**

**2020 50 20988 19855.422**

**2020 51 20462 20725.209**

**2020 52 21825 20087.398**
